# CSH RNA Interference Reduces Global Nutrient Uptake and Umbilical Blood Flow Resulting in Intrauterine Growth Restriction

**DOI:** 10.3390/ijms22158150

**Published:** 2021-07-29

**Authors:** Amelia R. Tanner, Cameron S. Lynch, Victoria C. Kennedy, Asghar Ali, Quinton A. Winger, Paul J. Rozance, Russell V. Anthony

**Affiliations:** 1 College of Veterinary Medicine, Colorado State University, Fort Collins, CO 80523, USA; amelia.tanner@colostate.edu (A.R.T.); cameronlynch553@gmail.com (C.S.L.); Tori.Kennedy@colostate.edu (V.C.K.); asghar.ali20@alumni.colostate.edu (A.A.); Quinton.Winger@colostate.edu (Q.A.W.); 2 Department of Pediatrics, University of Colorado Anschutz Medical Campus, Aurora, CO 80045, USA; paul.rozance@cuanschutz.edu

**Keywords:** chorionic somatomammotropin, blood flow, intrauterine growth restriction, nutrient uptake, uterus

## Abstract

Deficiency of the placental hormone chorionic somatomammotropin (CSH) can lead to the development of intrauterine growth restriction (IUGR). To gain insight into the physiological consequences of CSH RNA interference (RNAi), the trophectoderm of hatched blastocysts (nine days of gestational age; dGA) was infected with a lentivirus expressing either a scrambled control or CSH-specific shRNA, prior to transfer into synchronized recipient sheep. At 90 dGA, umbilical hemodynamics and fetal measurements were assessed by Doppler ultrasonography. At 120 dGA, pregnancies were fitted with vascular catheters to undergo steady-state metabolic studies with the ^3^H_2_O transplacental diffusion technique at 130 dGA. Nutrient uptake rates were determined and tissues were subsequently harvested at necropsy. CSH RNAi reduced (*p* ≤ 0.05) both fetal and uterine weights as well as umbilical blood flow (mL/min). This ultimately resulted in reduced (*p* ≤ 0.01) umbilical IGF1 concentrations, as well as reduced umbilical nutrient uptakes (*p* ≤ 0.05) in CSH RNAi pregnancies. CSH RNAi also reduced (*p* ≤ 0.05) uterine nutrient uptakes as well as uteroplacental glucose utilization. These data suggest that CSH is necessary to facilitate adequate blood flow for the uptake of oxygen, oxidative substrates, and hormones essential to support fetal and uterine growth.

## 1. Introduction

Intrauterine growth restriction (IUGR), the second-leading cause of perinatal mortality, results from a failure of the fetus to reach its growth potential in utero, and impacts up to 6% of pregnancies worldwide [1]. This pathology has also been linked epidemiologically to the development of adult-onset diseases such as cardiovascular disease, diabetes, and hypertension [2,3,4,5]. While many causes of IUGR remain unknown, deficiency of the placental hormone chorionic somatomammotropin (CSH) has been associated with fetal growth restriction in both humans [6,7] and sheep [8]. This relationship was directly demonstrated by our laboratory, utilizing lentiviral mediated RNA interference (RNAi) in the sheep placenta [9]. Using this model, CSH RNAi resulted in fetal and placental growth restriction in near-term (135 dGA) pregnancies [9], and by the end of the first third (50 dGA) of pregnancy [10]. However, CSH deficiency does not always result in IUGR, a phenomenon which is also documented in human pregnancies [6,11]. We have previously described this normal weight phenotype in response to CSH RNAi, which is characterized by decreased umbilical IGF1 [12], reduced uterine blood flow and increased placental glucose utilization [13]. These perturbations, in spite of normal fetal and placental weights, support the necessity of CSH for maintaining adequate uterine blood flow and regulating placental glucose consumption. Additionally, these CSH dependent effects hint at potential mechanisms that could be responsible for the development of IUGR in more severe CSH RNAi phenotypes. Thus, to better understand the actions of CSH in modulating fetal growth, our objective was to assess the physiological ramifications of CSH RNAi induced IUGR, to ascertain how CSH deficiency leads to the progression of fetal and placental growth restriction. We hypothesized that CSH RNAi induced IUGR results from altered uterine blood flow and nutrient uptake. It was determined that CSH RNAi resulted in decreased uterine and umbilical blood flows, reduced global nutrient transport and altered fetal IGF1 concentrations, driving the development of IUGR. 

## 2. Results

All equations used to calculate blood flow, nutrient uptake, nutrient utilization, and nutrient quotients are summarized in Table 1. 

### 2.1. 90dGA Doppler Velocimetry

**Figure 1:**
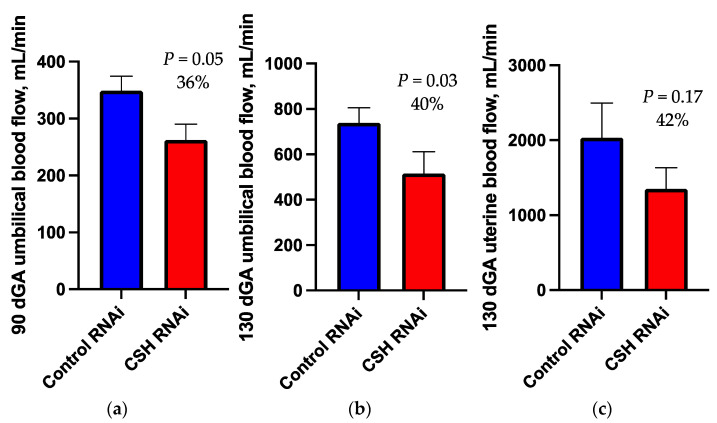
Measures of blood flow: (**a**) umbilical blood flow as assessed by Doppler ultrasound at 90 dGA; (**b**) umbilical blood flow as assessed by the transplacental diffusion technique at 130 dGA; (**c**) uterine blood flow as assessed by the transplacental diffusion technique at 130 dGA. Data are shown as means ± SEM for all pregnancies in each treatment group. CSH, chorionic somatomammotropin; RNAi, RNA interference.

As assessed by Doppler ultrasound and velocimetry, fetal binocular distance (cm), biparietal circumference (cm), abdominal circumference (cm), femur length (cm), and tibia length (cm) did not differ (*p* ≥ 0.10; Table 2) between treatments. Umbilical blood flow (mL/min) was reduced (*p* = 0.05; Figure 1) by 36% in CSH RNAi pregnancies. Umbilical artery cross sectional area (CSA, cm^2^) and cross-sectional diameter (CSD, cm) both tended (*p* ≤ 0.10; Table 2) to be reduced in CSH RNAi pregnancies. Resistance indices (RI), pulsatility indices (PI), systolic/diastolic ratios (S/D ratios), and fetal heart rate (bpm) did not differ between treatments.

### 2.2. 130dGA Uterine and Umbilical Blood Flows

As calculated by the transplacental diffusion technique, uterine blood (Figure 1) and plasma flows (mL/min; Table 2) in CSH RNAi pregnancies were not statistically different between treatments, nor were relative uterine blood or plasma flow (mL/min/kg fetus or mL/min/100 g placenta) different between treatments (Table 2). While caruncular endothelial nitric oxide synthase (NOS3) was numerically reduced by 38% in CSH RNAi pregnancies, this difference was not statistically significant (*p* = 0.38; Supplemental Figure S1).

Total umbilical (Figure 1) and plasma blood flows (mL/min) were reduced (*p* ≤ 0.05; Table 2) by 40% in CSH RNAi pregnancies. Umbilical blood flow relative to fetal or placental weight (mL/min/kg fetus and mL/min/100 g placenta) both tended to be reduced (*p* ≤ 0.10) in CSH RNAi pregnancies, but umbilical plasma flow relative to fetal weight was not different. Cotyledonary NOS3 was numerically elevated by 59% (*p* = 0.11; Supplemental Figure S1). Neither the uterine to umbilical blood flow ratio nor uterine and umbilical hematocrits were significantly altered by treatment. 

### 2.3. Fetal and Uteroplacental Characteristics Near-Term (130 dGA)

**Figure 2:**
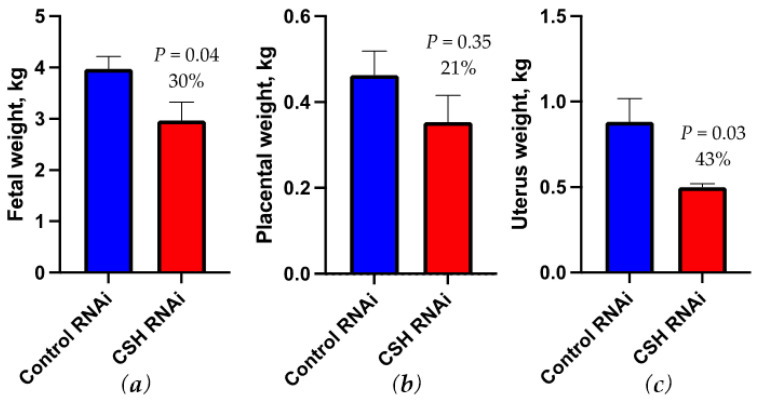
Measures of fetal and uteroplacental mass at 130 dGA: (**a**) fetal weight in kg (**b**) placental weight in kg; (**c**) uterus weight in kg. Data are shown as means ± SEM for all pregnancies in each treatment group. CSH, chorionic somatomammotropin; RNAi, RNA interference.

Fetal weight was reduced (*p* = 0.04; Figure 2) by 30% by CSH RNAi, but the fetal measurements (Table 3) of crown-rump length (cm) and ponderal index did not differ between treatments. Fetal hindlimb leg length (cm) however, was reduced (*p* = 0.02) in CSH RNAi fetuses. Fetal liver weights tended (*p* = 0.10; Table 3) to be reduced in CSH RNAi pregnancies, with right liver lobe mass tending to be lighter (*p* = 0.06) by 37%. Fetal heart weight was also numerically smaller by 23% (*p* = 0.11) in CSH RNAi fetuses, with left ventricular weights significantly reduced (*p* = 0.05) by 26%. Fetal brain weight did not differ (*p* = 0.68) between treatments, but brain weight relative to fetal weight was increased (*p* = 0.03) by 43% in CSH RNAi fetuses, an indicator of asymmetric fetal growth. Fetal muscle mass including the biceps femoris, soleus, flexor digitorum superficialis (FDS), tibialis anterior (TA), and extensor digitorum longus (EDL) were all reduced (*p* ≤ 0.05) in CSH RNAi fetuses. 

Placental weight of CSH RNAi pregnancies was not different from controls (Figure 2), however uterine weights were 43% smaller (*p* = 0.03; Figure 2) compared with control pregnancies. Uteroplacental weights also tended (*p* = 0.09; Table 3) to be reduced by 27% in CSH RNAi pregnancies. Fetal membrane weight and placentome number were not significantly altered by CSH RNAi. Cotyledonary CSH was numerically reduced by 36% in CSH RNAi placentae, but did not reach statistical significance (*p* = 0.17; Supplemental Figure S2).

### 2.4. Blood Gas and Oxygen Uptakes

**Figure 3:**
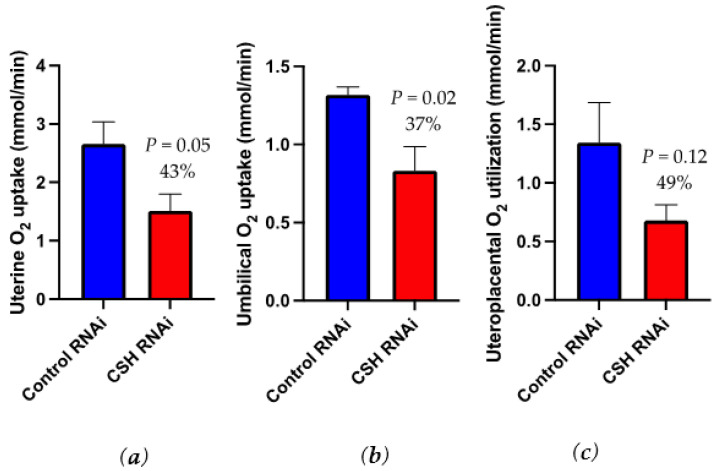
Uterine, umbilical, and uteroplacental oxygen uptakes as assessed by ^3^H_2_0 transplacental diffusion at 130 dGA: (**a**) uterine oxygen uptakes, mmol/min (**b**) umbilical oxygen uptakes, mmol/min; (**c**) uteroplacental oxygen utilization, mmol/min. Data are shown as means ± SEM for all pregnancies in each treatment group. CSH, chorionic somatomammotropin; RNAi, RNA interference.

As calculated by the ^3^H_2_0 transplacental diffusion technique, uterine oxygen uptake (mmol/min) was reduced (*p* = 0.05; Figure 3) by 43% in CSH RNAi pregnancies, but not on a relative basis (mmol/min/kg uterus; Table 4). Umbilical oxygen uptake (mmol/min) was reduced (*p* = 0.02; Figure 3) by 37% in CSH RNAi pregnancies, with relative (mmol/min/kg fetus) oxygen uptakes also tending (*p* = 0.07) to be suppressed. Uteroplacental oxygen utilization, both absolute (mmol/min; Figure 3) and relative (mmol/min/kg placenta; Table 4) were numerically lower (*p* = 0.12) in CSH RNAi pregnancies, but did not reach statistical significance. Uterine artery and vein blood gas and biochemistry measurements were not altered by CSH RNAi, (Supplemental Table S1), but both umbilical artery and vein oxygen content (O_2_ ct; mmol/L) tended to be reduced (*p* ≤ 0.10; Supplemental Table S2). 

### 2.5. Glucose and Lactate Uptakes

**Figure 4:**
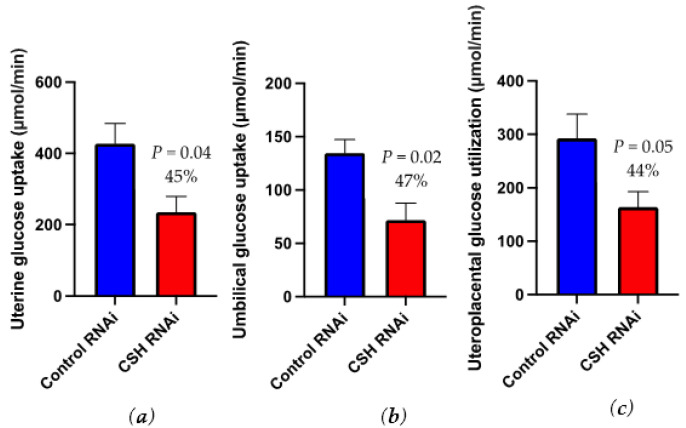
Uterine, umbilical, and uteroplacental glucose uptakes as assessed by ^3^H_2_0 transplacental diffusion at 130 dGA: (**a**) uterine glucose uptakes, μmol/min (**b**) umbilical glucose uptakes, μmol/min; (**c**) uteroplacental glucose utilization, μmol/min. Data are shown as means ± SEM for all pregnancies in each treatment group. CSH, chorionic somatomammotropin; RNAi, RNA interference.

Uterine glucose uptake (μmol/min) was reduced (*p* = 0.04; Figure 4) by 45% in CSH RNAi pregnancies but relative uterine glucose uptake (μmol/min/kg uterus; Table 4) was not changed by treatment. Umbilical glucose uptake (μmol/min; Figure 4) was suppressed (*p* = 0.02) by 47% in CSH RNAi fetuses, and by 27% (*p* = 0.06) relative to fetal weight (μmol/min/kg fetus). Uteroplacental glucose utilization (μmol/min) was 44% lower (*p* = 0.05; Figure 4) in CSH RNAi pregnancies whereas relative uteroplacental glucose utilization (μmol/min/kg placenta) was not statistically impacted (*p* = 0.15). On a placental weight basis, the quantity of glucose transferred to the fetus (μmol/kg placenta/min) tended (*p* = 0.10) to be reduced by 34% in CSH RNAi pregnancies. Due to the decrease in glucose uptake and utilization, we assessed the concentrations of the key placental glucose transporters, SLC2A1 and SLC2A3. The placental concentrations of SLC2A1 and SLC2A3 were not altered by treatment (Supplemental Figures S3 and S4). 

Uterine absolute (μmol/min; *p* = 0.22) and relative (μmol/min/kg uterus; *p* = 0.28) lactate secretions were not significantly impacted (Table 4) in CSH RNAi pregnancies. In contrast to control pregnancies which had positive uterine lactate secretion (Table 4), CSH RNAi pregnancies had negative uterine lactate secretion which indicates a net uptake of lactate by the uterine tissues. This was also reflected by negative uteroplacental utilization of lactate in CSH RNAi pregnancies (Table 4). In spite of limited uteroplacental lactate utilization, umbilical lactate uptake (μmol/min) was reduced (*p* = 0.03) by 39% in CSH RNAi fetuses.

### 2.6. Amino Acid Uptakes

**Figure 5:**
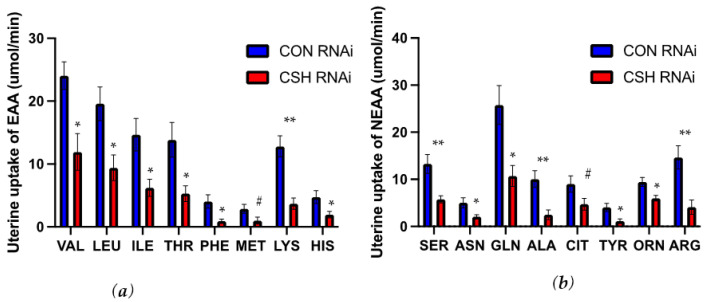
Uterine uptakes of essential (EAA) and nonessential (NEAA) assessed by ^3^H_2_0 transplacental diffusion at 130 dGA: (**a**) uterine uptakes of essential amino acids, μmol/min (**b**) uterine uptakes of essential amino acids, μmol/min. Data are shown as means ± SEM for all pregnancies in each treatment group. CSH, chorionic somatomammotropin; RNAi, RNA interference. Ala, alanine; Arg, arginine; Asn, asparagine; Cit, citrulline; Gln, glutamine; His, histidine; Ile, isoleucine; Leu, leucine; Lys, lysine; Met, methionine; Orn, ornithine; Phe, phenylalanine; Ser, serine; Thr, threonine; Tyr, tyrosine; Val, valine. ** *p* ≤ 0.01, * *p* ≤ 0.05, ^#^ *p* ≤ 0.10, when CSH RNAi pregnancies are compared with control RNAi pregnancies.

The uterine uptakes (μmol/min) of alanine, arginine, asparagine, glutamine, histidine, isoleucine, leucine, lysine, ornithine, phenylalanine, serine, threonine, tyrosine, and valine were all reduced (*p* ≤ 0.05; Figure 5) in CSH RNAi pregnancies, with the uptakes of both citrulline and methionine also tending (*p* ≤ 0.10; Figure 5) to be reduced. The relative uterine uptakes of alanine and lysine (μmol/min/kg uterus) were also reduced (*p* ≤ 0.05; Supplemental Table S3) in CSH RNAi pregnancies, while the relative uterine uptake of phenylalanine tended (*p* = 0.07) to be reduced.

**Figure 6:**
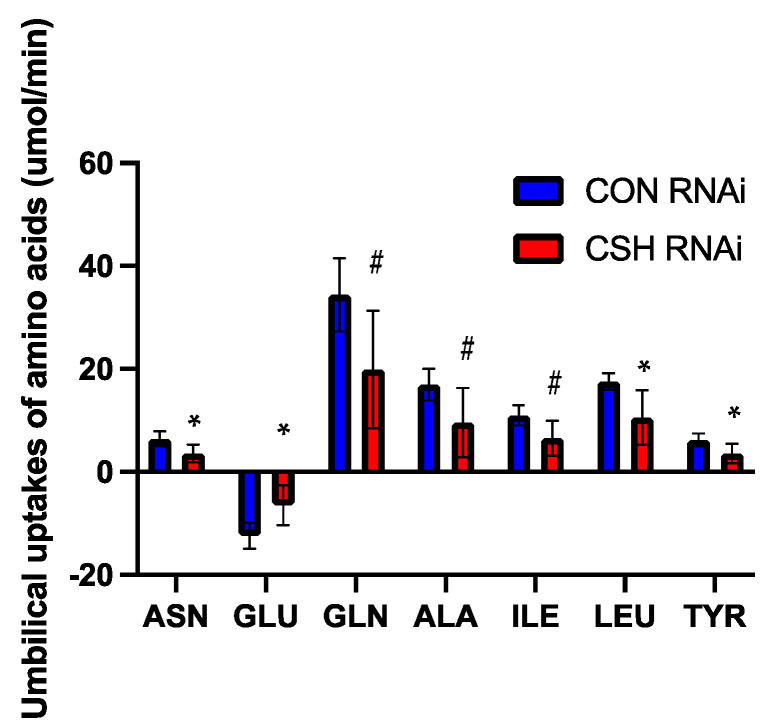
Umbilical uptakes (μmol/min) of amino acids as assessed by ^3^H_2_0 transplacental diffusion at 130 dGA. Data are shown as means ± SEM for all pregnancies in each treatment group. CSH, chorionic somatomammotropin; RNAi, RNA interference. Ala, alanine; Asn, asparagine; Glu, glutamate; Gln, glutamine; Ile, isoleucine; Leu, leucine; and Tyr, tyrosine. * *p* ≤ 0.05, ^#^ *p* ≤ 0.10, when CSH RNAi pregnancies are compared with control RNAi pregnancies.

Not only were the uterine uptakes of amino acids impaired in CSH RNAi pregnancies, but the umbilical uptakes (μmol/min) of asparagine, leucine, and tyrosine were also reduced, whereas the fetal production of glutamate was decreased (*p* ≤ 0.05; Figure 6). Additionally, the umbilical uptakes of glutamine, alanine, and isoleucine also tended (*p* ≤ 0.10; Figure 6) to be reduced in CSH RNAi fetuses. The relative fetal production of taurine (μmol/min/kg fetus) tended (*p* = 0.08; Supplemental Table S4) to be increased in CSH RNAi pregnancies whereas the relative umbilical uptake of asparagine tended (*p* = 0.09) to be reduced.

**Figure 7:**
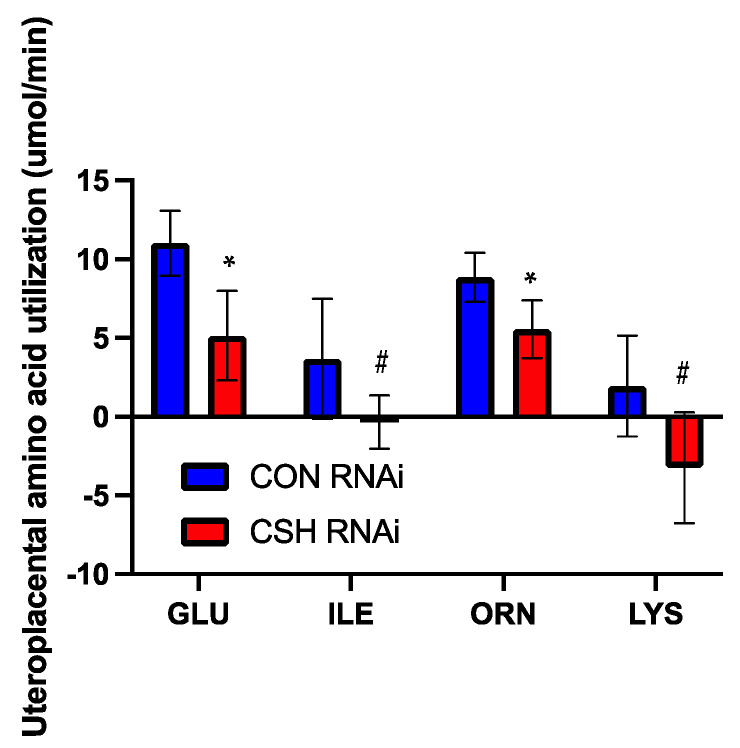
Uteroplacental utilization (μmol/min) of amino acids as assessed by ^3^H_2_0 transplacental diffusion at 130 dGA. Data are shown as means ± SEM for all pregnancies in each treatment group. CSH, chorionic somatomammotropin; RNAi, RNA interference. Glu, glutamate; Ile, isoleucine; Lys, lysine; and Orn, ornithine. * *p* ≤ 0.05, ^#^ *p* ≤ 0.10, when CSH RNAi pregnancies are compared with control RNAi pregnancies.

The placenta was also impacted by perturbed amino acid utilization in CSH RNAi pregnancies. The uteroplacental utilization (μmol/min) of glutamate and ornithine was reduced (*p* ≤ 0.05; Figure 7) in CSH RNAi pregnancies, whereas the uteroplacental utilization of isoleucine and lysine was negative (*p* ≤ 0.10). This response may indicate a lack of utilization to facilitate transfer to the fetus. On a relative basis (μmol/min/kg placenta), the uteroplacental utilization of lysine was negative (*p* = 0.03; Supplemental Table S5), again indicating a lack of utilization to facilitate transfer to the fetus. Additionally, the uteroplacental utilization of glutamate and isoleucine tended (*p* ≤ 0.10) to be reduced in CSH RNAi pregnancies. 

### 2.7. Total Nutrient Uptakes

With the transplacental diffusion technique, it is possible to calculate substrate specific carbon supply to both the uterus and the fetus (Table 1). Furthermore, it is possible to calculate the total carbon and nitrogen available for catabolic processes. As summarized in Supplemental Table S6, uterine carbon uptakes from amino acids (*p* = 0.004), glucose (*p* = 0.04), and lactate (*p* = 0.22) were reduced in CSH RNAi pregnancies. This resulted in a 60% reduction (*p* = 0.04) in total carbon available for uptake in CSH RNAi pregnancies. Uterine nitrogen uptake was also reduced (*p* = 0.001) by 61% in CSH RNAi pregnancies. 

Umbilical carbon uptakes from amino acids (*p* = 0.13; Supplemental Table S6), glucose (*p* = 0.02), and lactate (*p* = 0.03) were reduced in CSH RNAi fetuses leading to a 42% reduction (*p* = 0.04) in total carbon uptake. Umbilical nitrogen uptake was numerically lower (*p* = 0.13) in CSH RNAi fetuses. No umbilical nutrient to oxygen quotients differed between treatments (Supplemental Table S6). 

### 2.8. 130dGA Hormones

Uterine concentrations of insulin, insulin-like growth factor 1 (IGF1), cortisol, and estradiol-17β were not impacted by RNAi treatment, as summarized in Table 5. In contrast, umbilical artery IGF1 was reduced (*p* ≤ 0.01; Table 5) by 48% in CSH RNAi fetuses. Umbilical artery concentrations of insulin, cortisol and estradiol were not statistically impacted by CSH RNAi. 

## 3. Discussion

To describe the progression of CSH RNAi induced IUGR, we set out to document the physiological ramifications of CSH RNAi in a cohort of IUGR pregnancies. We hypothesized that CSH RNAi would reduce both fetal and maternal blood flow, resulting in reductions in global nutrient uptake. Unfortunately, due to fetal demise and catheter failures, complete studies were only accomplished on *n* = 4 pregnancies per group, somewhat limiting our statistical power. Additionally, while fetal sex was equally distributed (one female, three males) in each treatment, fetal sex could not be included in our statistical model. Regardless, results from this study directly support this hypothesis. 

By using Doppler ultrasonography, we established that by 90 dGA, umbilical blood flow was reduced in CSH RNAi pregnancies. It is somewhat surprising, however, that the decrease in blood flow was not accompanied by a corresponding increase in vascular resistance, a hallmark of placental insufficiency [14,15]. This might suggest that CSH is not acting in a way that reduces placental vascularity, which would have likely resulted in increased vascular resistance, but in a way that modulates the size of major vessels such as the umbilical artery, which was reduced in our current study. While not statistically different (*p* = 0.11), the ~60% increase in cotyledonary NOS3 in the CSH RNAi near term (130 dGA) can perhaps, in part, explain why there was not an increase in vascular resistance. This is supported by an ex vivo study of placental perfusion from human fetal growth-restricted pregnancies, where flow mediated vasodilation was reduced but NOS3 was still elevated [16]. This combination of normal vascular resistance with decreased umbilical blood flow appears to be a unique distinction between CSH RNAi induced IUGR and other forms of placental insufficiency investigated in sheep. For example, in hyperthermia induced IUGR pregnancies, umbilical blood flow is reduced, and vascular resistance is increased [14,17]. Those pregnancies also have reduced umbilical artery NOS3 mRNA concentrations [18].

Near term (130 dGA) uterine and umbilical blood flow was assessed by the ^3^H_2_0 transplacental diffusion technique. Total umbilical blood and plasma flows were significantly reduced in CSH RNAi pregnancies, as well as relative umbilical blood flows. This suggests the decrease in umbilical blood flow was somewhat independent of both fetal weight and placental weight. While the reduction (42%) in uterine blood flow in CSH RNAi pregnancies was not statistically different, it agrees with the statistically significant reduction in uterine blood flow reported earlier for CSH RNAi pregnancies [13]. In that study [13], uterine blood flow was reduced by only 24% with no change in uterine or fetal weights. Perhaps one driver of CSH RNAi induced IUGR is decreased uterine blood flow, which precedes a subsequent uterine mass reduction and ultimately prevents the uteroplacental unit from transporting adequate nutrients to sustain fetal growth.

With the noted decreases in blood flow, it is not surprising that fetal weights were reduced in this cohort of CSH RNAi pregnancies. Compared to a previous study that also reported near-term IUGR in CSH RNAi pregnancies, a similar degree of fetal growth restriction was observed with a 30% weight reduction in the current cohort vs. 32% in the Baker et al. [9] study. It appears that CSH RNAi induced IUGR can be classified as asymmetric IUGR as the brain to fetal weight ratio was significantly elevated, a proven indicator of asymmetric fetal growth [19]. This cohort of CSH RNAi pregnancies with IUGR also had reduced lower leg length, which was also observed in CSH RNAi pregnancies with normal fetal weights [13]. 

Similar to fetal weight reductions, the fetal livers of CSH RNAi pregnancies in this study were comparably reduced (35% vs. 36.5%) as in the Baker et al. [9] study, suggesting the necessity of CSH in preserving liver mass and corresponding liver-mediated IGF1 production. A novel finding reported on this cohort of CSH RNAi pregnancies was proportionately smaller hearts, especially the left ventricles. In a sheep model of experimentally induced placental growth restriction (carunclectomy), left ventricular cardiomyocytes number was reduced in the offspring during adulthood, and was correlated to birth weight [20]. Fetal muscle weights were also significantly reduced in this CSH RNAi IUGR cohort, as has been documented in other sheep models of IUGR [21,22]. In this current study, this effect does appear to be a function of fetal weight reduction, as normalizing each muscle relative to fetal mass removes the differences except for the flexor digitorum superficialis. 

In the current cohort, there was a 21% reduction in placental weight, which wasn’t statistically different from the control pregnancies. While it can certainly be argued that CSH RNAi did induce metabolic, hemodynamic and growth perturbations to these pregnancies, it is also accurate that this was a less severe placental growth restriction compared to the 52% placental mass reduction in the Baker et al. [9] study. While difficult to truly assess the differences between the two studies, one possible explanation includes variability of robustness in the activity of the RNAi over gestation. If the RNAi is more robustly expressed during key timepoints in placentation, it is possible that the degree of placental mass reduction would be more severe. Based on placental efficiency, the smaller placentas in the Baker et al. study [9] could be argued to be more efficient, as they produced approximately the same degree of fetal growth restriction as the current study. Thus, it is possible that the impacts of CSH RNAi yield consistent degrees of reduced fetal growth in the IUGR phenotype, which could suggest similar placental dysfunction between the two studies. 

One truly novel insight into the current cohort provided was the potential impacts of CSH on uterine size, as it was not assessed in the Baker et al. [9] study. There is some evidence during early pregnancy that CSH can impact the uterus. In ovariectomized ewes that have been hormonally manipulated to mimic early pregnancy, CSH infusion acts on the endometrial glands [23]. Through the intrauterine infusion of exogenous recombinant CSH from 16–25 days post estrus, endometrial gland density increased [23]. A similar effect occurred when a separate treatment of growth hormone (GH) was infused over the same timeframe, suggesting that both CSH and GH may act on the early-pregnancy endometrium in a similar fashion [23]. This also may suggest a potential role for CSH, working through the same receptor to support uterine modifications for successful pregnancy outcomes. 

The direct vs. indirect impacts of CSH on uterine mass are quite difficult to tease apart. In sheep, pregnancy increases nitric oxide, which has been shown to stimulate uterine blood flow and increase NOS3 in the uterine vascular endothelium [24]. As reported by Tanner et al. [13], CSH RNAi results in similar reductions in both uterine blood flow (24%) and caruncular NOS3 (24%), even in the absence of IUGR or uterine weight reductions. In the present cohort of CSH RNAi induced IUGR pregnancies, similar degrees of reductions in uterine blood flow (42%) and NOS3 (38%) were also observed. It is plausible that this relationship could influence the perfusion of the uterus, partially explaining the reduced uterine weights. This is supported by the global decrease in the uterine uptakes of oxygen, glucose, and amino acids. 

Through the application of the ^3^H_2_0 transplacental diffusion technique, the uptake of substrates from maternal circulation and their partitioning to uterus, fetus and placenta can be directly assessed. Pregnancies perturbed by IUGR often experience global reductions in the uptakes of all of the key substrates including oxygen, glucose, and amino acids. This study again demonstrated this relationship with global reductions in the uptakes of all three substrates by all aspects of uteroplacental circulation. It is worth noting that the uterine uptakes of substrates (Table 1) really represent the quantity of substrate the entire uteroplacental unit (uterus and placenta) is taking up from maternal circulation. When the umbilical uptake of the substrate is accounted for, the difference between uterine (uteroplacental) and umbilical uptakes represents what the uteroplacental unit is utilizing. A novel but not entirely unexpected finding is the global reduction in oxygen uptake by the CSH RNAi induced IUGR pregnancies. Oxygen uptake is considered a flow limited substrate [25,26] and blood flow was reduced in this cohort of CSH RNAi induced IUGR pregnancies. Other sheep models of placental insufficiency IUGR are also characterized by uteroplacental reductions in oxygen uptakes [19,27]. Kingdom and Kaufmann [28] described several modalities for examining pregnancy hypoxia and their respective etiologies. It appears that CSH deficiency results in placental hypoxia, where maternal blood is adequately oxygenated (as evidenced by no differences in maternal oxygen content, see Supplemental Table S1) but oxygen cannot adequately diffuse across the placenta [28], as evidenced by reduced fetal uptakes and uteroplacental utilization. In this case, it appears that reduced oxygen uptake is likely originating from reduced blood flow, resulting in a smaller uterine mass which cannot transport as much oxygen from maternal circulation. Functionally, this means both the fetus and placenta had less oxygen available for oxidative metabolism and, not surprisingly, their growth was reduced. 

While we have previously demonstrated that CSH RNAi results in reductions to the fraction of glucose transferred to the fetus, even in cases of normal fetal weights [13], the effects on glucose uptake in this cohort of CSH RNAi induced IUGR pregnancies were far more severe. As glucose is considered the primary substrate for fetal oxidative metabolism in both humans and sheep [29], it is not surprising that reductions in glucose uptake and utilization by the uteroplacental unit and the fetus in this cohort led to IUGR. Even when umbilical glucose uptake was normalized to fetal weight, glucose uptake was still reduced, suggesting a direct impact of CSH on umbilical glucose uptake as it is not just a function of fetal mass. CSH RNAi also reduced umbilical lactate uptake, leading to further reductions in carbon sources for the fetus. 

Placental glucose transport is dependent on several factors, including the maternofetal glucose gradient, the availability of transporters to shuttle glucose across the placenta, the oxidative needs of the placenta, and placental mass [25,30]. In the current study, there were no significant changes to placental SLC2A1 or SLC2A3 concentrations. This fits with the lack of SLC2A1 changes in the human placentae of IUGR pregnancies [31]. Ultimately, this suggests that the reductions in glucose uptake and utilization by the uteroplacental unit are likely due to decreases in blood flow and uteroplacental mass. 

One clear distinction between IUGR and normal weight CSH RNAi phenotypes is the impact on uteroplacental (placental) glucose utilization. In CSH RNAi pregnancies with normal fetal weights, uteroplacental glucose utilization was increased but the fetal uptakes of glucose were not altered [13]. This suggests that with reduced CSH, the placenta requires additional glucose to support appropriate fetal growth and transfer of nutrients to the fetus. This might hint at a potential compensatory mechanism in the normal weight pregnancies that was not sufficient to maintain fetal growth in our CSH RNAi IUGR pregnancies. In the more severe IUGR CSH RNAi phenotype, it is likely that this compensatory mechanism is overridden, possibly being preceded by greater proportional reductions in blood flow leading to a smaller uterus, unable to transfer enough glucose from maternal circulation to support adequate placental function and, therefore, fetal growth. 

Amino acids are also considered an important oxidative substrate for fetal development [25]. In the current cohort of CSH RNAi pregnancies with IUGR, the uterine uptakes of eight essential amino acids (including all three branch-chain amino acids) were reduced along with eight nonessential amino acids. This resulted in substantial reductions in carbon (60%) and nitrogen (61%) taken up by the uterus (see Supplemental Table S6). This could contribute to the explanation of why the uteri containing CSH RNAi pregnancies were smaller. In normal weight CSH RNAi pregnancies, only the uterine uptake of taurine and glycine were reduced [13]. It is interesting that neither of these amino acids were statistically reduced in CSH RNAi IUGR pregnancies. Regardless of these variations, it does appear that uterine uptakes of amino acids were more profoundly impacted in cases of CSH RNAi IUGR. 

The decreased uterine uptakes of amino acids also resulted in fewer amino acids available for fetal uptake in CSH RNAi pregnancies, with reduced uptakes of both isoleucine and leucine and five non-essential amino acids, including glutamine. Because the concentration of amino acids is usually higher in fetal circulation, the transport of amino acids is dependent on energy availability due to active transport mechanisms necessary to transport against the concentration gradient [32]. In CSH RNAi pregnancies with IUGR, there was reduced placental utilization of both glutamate and ornithine, as well as limited uteroplacental utilization of isoleucine and lysine. In both sheep and humans, there is no net placental transfer of glutamate [33,34]. The fetus takes up glutamine from uterine circulation, and the fetal liver converts it to glutamate to supply the placenta [35]. Thus, the decreased utilization of glutamate by the CSH RNAi uteroplacental unit is likely due to the decreased umbilical uptakes of glutamine, leading to the reduced production of glutamate from by the fetal liver.

One of the key postulated roles for CSH in regulating fetal growth is the stimulation of the insulin-like growth factors (IGFs) [36]. This is supported by suggestive evidence in humans of parallel increases in IGF1 and CSH in maternal circulation [37]. In hypophysectomized rats, ovine CSH was infused which stimulated IGF1 secretion [38]. Furthermore, in pregnant rats and sheep, plasma IGF1 concentrations are maintained after hypophysectomy until delivery of the placenta [36,39]. Based on that evidence, Handwerger hypothesized that CSH stimulated IGF1 in both maternal and fetal circulation [36]. Interestingly, in our CSH RNAi pregnancies [9,12,13], maternal concentrations of IGF1 were not influenced by CSH RNAi, which differs from what was originally hypothesized. 

However, our data does support the proposed actions of CSH on IGF1 secretion in the fetus. Handwerger suggested CSH likely stimulated fetal IGF1 and IGF2 production, as evidenced by the treatment of rat embryonic fibroblasts with ovine CSH [40]. We have directly and repeatedly demonstrated that CSH RNAi causes reductions in umbilical IGF1 concentrations, in both the IUGR ([9] and the current cohort) and normal weight phenotypes [12]. Furthermore, CSH is also likely acting in a paracrine fashion on the placenta to impact placental IGFs, as evidenced by CSH RNAi reducing placental mRNA concentrations of IGF1 and IGF2 [10]. This is relevant to the current study, as one of the roles of the IGFs includes the placental transport of glucose and amino acids [41]. Because of the direct effects of CSH on the secretion of IGF1 in fetal circulation, it is not surprising that CSH RNAi results in reduced placental glucose and amino acid transfer, as well as subsequent IUGR. 

The IGFs are not the only hormone postulated to be impacted by CSH. CSH has been postulated to increase maternal insulin secretion and impair glucose tolerance [36]. Ex vivo, CSH has been demonstrated to stimulate insulin secretion by isolated pancreatic islet cells [42]. However, our in vivo data do not support this, as in both the CSH RNAi IUGR ([9] and current study) and normal weight phenotypes [12], maternal circulating insulin concentrations are not altered. Furthermore, maternal glucose concentrations are not impacted by CSH RNAi in either the IUGR (current study) or normal weight [13] phenotypes. Maternal glucose and insulin concentrations were also unaltered in a model of CSH infusion into the late gestation sheep [43]. In the fetus, however, CSH does appear to contribute to insulin concentration. In previous [9] and current cohorts of CSH RNAi fetuses with IUGR, umbilical insulin concentrations were reduced by 48% and 39%, respectively. It would be very interesting to follow a cohort of CSH RNAi offspring into adulthood to examine adult glucose homeostasis and insulin sensitivity, as CSH RNAi results in both hypoglycemia and likely hypoinsulinemia. Overall, our in vivo data support the postulated roles of CSH in fetal circulation, but not maternal.

## 4. Materials and Methods

All animal procedures were approved by the Colorado State University Institutional Animal Care and Use Committee (Protocol # 18-7866A), the Institutional Biosafety Committee (18-029B) and the University of Colorado Anschutz Medical Campus Institutional Animal Care and Use Committee (Protocol #00714).

### 4.1. Lentiviral Generation

Lentiviral generations of hLL3.7 tg6 (target 6; CSH RNAi) and hLL3.7 NTS (scramble control/non-targeting sequence; control RNAi) are summarized in Supplemental Table S7 as described previously [9,10,13]. Briefly, both the NTS and tg6 sequences [9,10,13] were cloned into the LL3.7 vector, and all subsequent virus generation and titration were completed in accordance with our previously described procedures [9].

### 4.2. Generation of CSH RNAi Pregnancies 

All ewes (Dorper breed composition) were group housed in pens at the Colorado State University Animal Reproduction and Biotechnology Laboratory, and were provided access to hay, trace mineral, and water in order to meet or slightly exceed their National Research Council [44] requirements. All animal procedures were conducted as previously described [9,10,13]. In summary, after synchronization and subsequent breeding, fully expanded and hatched blastocysts were collected by flushing the uteri at 9 days post conception. Each blastocyst was infected with 100,000 transducing units of either NTS/control RNAi or CSH RNAi virus. After infection for approximately 5 hours, each blastocyst was washed and a single blastocyst was transferred surgically [9,10,13] into a synchronized recipient ewe. Each recipient ewe was monitored daily for return to standing estrus, and confirmed pregnant at 50 days of gestational age (dGA) by ultrasound (Mindray Medical Equipment, Mahwah, NJ, USA). Using these methods, 6 Control RNAi and 6 CSH RNAi pregnancies were generated. 

### 4.3. Doppler Velocimetry 

At 90 dGA, all pregnancies (*n* = 12) underwent Doppler velocimetry using a Mindray M7 Premium (Mindray) with a convex C5-2s probe (2.1–5.1 MHz) abdominal transducer by a single technician. All ewes were examined supine in a recumbent position. After confirming fetal viability, fetal binocular distance (cm), biparietal circumference (cm), abdominal circumference (cm), femur (cm) and tibia length (cm) were recorded and averaged across 3 independent measurements. Pulse-waved Doppler velocimetry measurements of the umbilical artery were performed with color flow Doppler with an angle of insonation of 30 degrees [14,17]. Doppler indices of perfusion including pulsatility indices (PI; Table 1) and resistance indices (RI; Table 1), as well as a systolic:diastolic ratio (S/D), were recorded on 3 independent sets of 3 cardiac cycles (minimum of 9 cardiac waveforms) and averaged [14,17]. Fetal heart rate was also recorded as a 3 cardiac cycle average. Umbilical blood flow (mL/min) was calculated by time-averaged mean velocity (cm/s) × π/4 × vessel cross-sectional area (cm^2^) × 60. One control RNAi ewe had umbilical hemodynamic measurements excluded due to failure to achieve a 30-degree angle of insonation, but all fetal measurements from that animal were included.

### 4.4. Surgical Instrumentation of Fetus and Ewe

At approximately 115 dGA, pregnant recipient ewes were transported to the University of Colorado Anschutz Medical Campus, Perinatal Research Center (Aurora, CO, USA). Animals had access to ad libitum alfalfa pellets (Standlee Hay, Kimberly, ID) and water. All animals (6 = control RNAi and 6 = CSH RNAi pregnancies) underwent surgical placement of fetal and maternal catheters at 126 dGA, to determine blood flow and nutrient flux as previously described [13,19,45,46,47,48]. Briefly, the following catheters were placed: fetal descending aorta (representing umbilical artery blood), fetal femoral vein and umbilical vein, maternal femoral artery (representing uterine artery blood), maternal femoral vein, and uterine vein. Due to two fetal demises (one CSH RNAi and one control RNAi) and catheters failing to draw on the study day, a total of four control RNAi and four CSH RNAi animals completed the full study and were included in the final analysis. Fetal sex was balanced between both treatments, with 3 males and 1 female in each treatment group. 

### 4.5. Blood Flow Calculations and Tissue Collection

At 130 dGA, uterine and umbilical blood flows were determined by the steady state ^3^H_2_O transplacental diffusion technique, as summarized previously [13]. Briefly, samples were simultaneously collected from the maternal femoral artery (A), uterine vein (V), umbilical vein (γ) and fetal descending aorta (α) every 20 minutes and averaged across 4 draws for analysis of blood biochemistry, nutrient content, ^3^H_2_O and hormone concentrations [13]. 

As described extensively in Table 1, uterine and umbilical blood flows were calculated by the transplacental diffusion technique described previously [13,49,50]. Uterine, umbilical, and uteroplacental utilization of oxygen, glucose, lactate and amino acids were calculated by the transplacental diffusion technique [51] and reported as an average of draws one through four. All other calculations were described extensively in Tanner et al. [13]. 

Ewes and fetuses were euthanized, and tissues were harvested at 130 dGA as previously described by Tanner et al. [13]. Briefly, after trimming, placentomes were selected from each placenta and separated into cotyledonary (fetal) and caruncular (maternal) components, then snap frozen in liquid N_2_ and stored at −80 °C. Fetal weight and dissected organ weights were recorded, and tissues were snap frozen in liquid nitrogen. Ponderal index and fetal brain:liver weight ratios were calculated, as previously described [13].

### 4.6. Biochemical Analysis of Blood Samples

Whole blood O_2_ content, hemoglobin O_2_ saturation (SO_2_) partial pressure of oxygen (PO_2_), partial pressure of carbon dioxide (PCO_2_), pH and hematocrit measurements were analyzed by an ABL 800 Blood Gas analyzer, as previously described [13]. Plasma glucose and lactate were measured by Yellow Springs Instrument 2900 (YSI Incorporated, Yellow Springs, OH, USA), as described previously [13]. Plasma amino acids were measured by HPLC [13]. Maternal (uterine) and fetal (umbilical) concentrations of insulin, IGF1, and cortisol were assessed by enzyme-linked immunosorbent assay (ALPCO Immunoassays 80-INSOV-E01, 22-IGFHU-E01, and 11-CORHU-E01-SLV, respectively), as described previously [50,52,53]. Estradiol was analyzed by radioimmunoassay, as described by Gonzalez-Padilla et al. [54]. 

### 4.7. Western Blot Analysis

Protein isolation and analysis was conducted in accordance with methods described previously [13]. Cotyledonary or caruncular tissue (100 mg) was lysed in 500 µL of lysis buffer and sonicated on ice. For analysis of cotyledonary CSH, 5 µg of protein was electrophoresed through a 4–15% Tris-Glycine Stain-Free gel (BioRad, Hercules, CA, USA) and transferred via a Trans-Blot Turbo semi-dry transfer system (Bio-Rad) to a 0.20-µm pore nitrocellulose membrane. Total protein per lane was visualized using the ChemiDoc XRS+ chemiluminescence system (BioRad) to use for normalization. To visualize CSH, the blot was incubated in a 1:25,000 dilution (in 5% Non-Fat Dry Milk / 1X Tris-Bis Solution+ 1% Tween) of rabbit α- oPL-S4 [55] for 24 h at 4 °C. After washing the membrane, the membrane was transferred into a 1:5000 dilution (in 5% Non-Fat Dry Milk / 1X Tris-Bis Solution+ 1% Tween) of mouse α-rabbit IgG conjugated to horse radish peroxidase (ab-97051; Abcam, Cambridge, MA, USA). Nitrocellulose membranes were developed using an ECL Western Blotting Detection Reagent chemiluminescent kit (Amersham, Pittsburgh, PA) and imaged using the ChemiDoc XRS+ chemiluminescence system. Densitometry calculations were performed using Image Lab Software (BioRad). 

For analysis of caruncular and cotyledonary concentrations of NOS3, 20 µg of each sample was electrophoresed through 4–15% Tris-glycine stain-free gels (Bio-Rad), transferred and analyzed as described for CSH. NOS3 was detected using a 1:2000 dilution of mouse α-NOS3 (BD 610297; BD Biosciences, San Jose, CA) and a 1:5000 dilution of goat α-mouse IgG conjugated to horseradish peroxidase (sc-2005; Santa Cruz Biotechnology Inc., Dallas, TX). For analysis of caruncular and cotyledonary (5 µg/sample) concentrations of SLC2A1 (GLUT1), samples were electrophoresed, transferred, and analyzed as described above. SLC2A1 was detected using a 1:40,000 dilution of rabbit α-SLC2A1 (07–1401; EMD Millipore) and a 1:80,000 dilution of goat α-rabbit IgG conjugated to horseradish peroxidase (ab205718; Abcam). As described above, densitometry of SLC2A1 was normalized on total protein/lane. Analysis of SLC2A3 (GLUT3) was conducted as previously detailed in Tanner et al. [13]. Briefly, 10 µg samples of caruncular or cotyledonary tissue were electrophoresed through NuPAGE 4–12% Bis-Tris Gels (Life Technologies), transferred to nitrocellulose, and the resulting blots were stained with Ponceau S to assess total protein per lane using the ChemiDoc XRS+ (Bio-Rad). Subsequent procedures were as described above, using a 1:1000 dilution of CSU-α-SLC2A3-22, and a 1:5000 dilution of a goat α-rabbit IgG conjugated to horseradish peroxidase (ab97051; Abcam). All densitometry was conducted in accordance with the description above. 

### 4.8. Statistical Analysis

Data were analyzed by Student’s t-test in GraphPad Prism (8.3.1). Due to the limited number of females in each treatment group (*n* = 1), fetal sex x treatment interactions were not examined. Statistical significance was set at *p* ≤ 0.05 and a statistical tendency at *p* ≤ 0.10. Data are reported as the mean ± standard error of the mean (SEM).

## 5. Conclusions

While CSH has been previously demonstrated as necessary for adequate fetal growth, the specific role of CSH in modulating fetal growth has yet to be determined. In this current study, we examined the physiological ramifications of CSH deficiency in cases of IUGR during late gestation. These data suggest that CSH is not only important for uterine blood flow and uteroplacental glucose utilization, but it also facilitates adequate umbilical blood flow necessary for the uptakes of oxygen, oxidative substrates, and hormones necessary to support fetal growth. Additionally, the current study demonstrated a novel role of CSH is supporting uterine growth to facilitate adequate transfer of nutrients.

## Figures and Tables

**Table 1:** Calculations ^1^ for blood flow, nutrient uptake, nutrient utilization, and quotients.

Blood Flow (Doppler Ultrasound)	
Pulsatility index	(PSV–EDV)/Timed-average mean velocity (TAMV)
Resistance index	(PSV-EDV)/PSV
Umbilical Blood Flow (mL/min)	TAMV × (π/4) × Artery cross-sectional area × 60
**Blood Flow (^3^H_2_O Tracer)**	
R_inf_ ^3^H_2_O (dpm/min)	Pump rate × [infusate]
R_acc(f)_ (dpm/min)	αpl slope × (0.8 × fetal weight)
R_acc(m)_ (dpm/min)	R_acc(f)_ + [αpl slope × 0.8(uterine weight)]
Umbilical Blood Flow (UBF; mL/min)	(R_inf_ − R_acc(f))_/([^3^H_2_O]_α(WB)_ − [^3^H_2_O]_γ(WB)_)
Umbilical Plasma Flow (UPF; mL/min)	UBF × [1 − Hct_f(avg)_]
Uterine Blood Flow (UtBF; mL/min)	(R_inf_ − R_acc(m)_)/([^3^H_2_O]_V(WB)_ − [^3^H_2_O]_A(WB)_)
Uterine Plasma Flow (UtPF; mL/min)	UtBF × [1 − Hct_m(avg)_]
**Nutrient Uptake and Utilization Rates**	
Umbilical Oxygen Uptake (UOU; mmol/min)	UBF × ([O_2_]_γ(WB)_ − [O_2_]_α(WB)_)
Uterine Oxygen Uptake (UtOU; mmol/min)	UtBF × ([O_2_]_A(WB)_ − [O_2_]_V(WB)_)
Uteroplacental Oxygen Utilization (mmol/min)	UtOU − UOU
Plasma to WB Glucose Conversion	[G]_pl_ × [1 − (0.24 × Hct)] − (3.3 × Hct)
Umbilical Glucose Uptake (UGU; µmol/min)	UBF × ([G]_γ(WB)_ − [G]_α(WB)_)
Uterine Glucose Uptake (UtGU; µmol/min)	UtBF × ([G]_A(WB)_ − [G]_V(WB)_)
Uteroplacental Glucose Utilization (µmol/min)	UtGU − UGU
Umbilical Lactate Uptake (µmol/min; ULU)	UBF × ([L]_γ(pl)_ − [L]_α(pl)_)
Uterine Lactate Secretion (µmol/min; UtLS)	UtBF × ([L]_V(pl)_ − [L]_A(pl)_)
Uteroplacental Lactate Production (µmol/min)	ULU + UtLS
Umbilical AA Uptake (µmol/min; UAAU)	UPF × ([AA]_γ(pl)_ − [AA]_α(pl)_)
Uterine AA Uptake (µmol/min; UtAAU)	UtPF × ([AA]_A(pl)_ − [AA]_V(pl)_)
Umbilical AA Carbon Uptake (µmol/min; UCU)	(#AA carbons) × UAAU
Uterine AA Carbon Uptake (µmol/min; UtCU)	(#AA carbons) × UtAAU
Umbilical AA Nitrogen Uptake (µmol/min; UNU)	(#AA nitrogens) × UAAU
Uterine AA Nitrogen Uptake (µmol/min; (UtNU)	(#AA nitrogens) × UtAAU
**Fetal Nutrient: Oxygen Quotients**	
Glucose:Oxygen (G:O) quotient	6 × ([G]_γ(WB)_ − [G]_α(WB)_)/([O_2_]_γ(WB)_ − [O_2_]_α(WB)_)
Lactate:Oxygen (L:O) quotient	3 × ([L]_γ(pl)_ − [L]_α(pl)_)/([O_2_]_γ(WB)_ − [O_2_]_α(WB)_)
Amino Acid:Oxygen (AA:O) quotient	Q × ([AA]_γ(pl)_ − [AA]_α(pl)_)/([O_2_]_γ(WB)_ − [O_2_]_α(WB)_)
Total Nutrient:Oxygen quotient	G:O quotient + L:O quotient + Total AA:O quotient

^1^ Calculations are derived from Cilvik et al., 2021.

**Table 2:** Measures of blood flow and fetal growth as assessed by 90 dGA Doppler velocimetry and 130 dGA ³H_2_0 transplacental diffusion.

	CON RNAi	CSH RNAi	% Change	*p*-Value
90 dGA Doppler Ultrasound Measurements	(*n* = 6)	(*n* = 6)		
Binocular distance, cm	4.91 ± 0.25	4.63 ± 0.19	5.70	0.48
Biparietal circumference, cm	16.56 ± 0.63	15.11 ± 0.54	8.72	0.19
Abdominal circumference, cm	22.03 ± 0.95	20.30 ± 1.27	7.82	0.40
Femur length, cm	4.24 ± 0.08	4.17 ± 0.09	1.81	0.62
Tibia length, cm	3.05 ± 0.11	3.03 ± 0.04	0.75	0.87
Pulsatility Index	1.98 ± 0.16	2.04 ± 0.14	2.88	0.84
Resistance Index	0.69 ± 0.04	0.70 ± 0.02	1.12	0.89
Systolic:Diastolic	3.63 ± 0.34	3.59 ± 0.28	0.97	0.95
Fetal heart rate, bpm	190.48 ± 3.38	199.81 ± 8.91	4.90	0.48
Umbilical artery cross-sectional area, cm^2^	0.24 ± 0.02	0.18 ± 0.02	25.23	0.09
Umbilical artery cross-sectional diameter, cm	0.55 ± 0.02	0.47 ± 0.03	15.31	0.08
**130 dGA Transplacental Diffusion Blood Flow Measurements**	**(*n* = 4)**	**(*n* = 4)**		
Uterine plasma flow (mL/min)	1417.74 ± 328.48	807.99 ± 208.40	43.01	0.17
Relative uterine blood flow (mL/min/kg fetus)	500.07 ± 86.44	400.98 ± 59.35	19.81	0.38
Relative uterine plasma flow (mL/min/kg fetus)	348.20 ± 61.93	274.78 ± 41.11	21.09	0.36
Uterine blood flow/100 g placenta	429.05 ± 54.03	320.80 ± 59.32	25.23	0.23
Umbilical plasma flow (mL/min)	490.84 ± 52.03	293.18 ± 63.87	40.27	0.05
Relative umbilical blood flow (mL/min/kg fetus)	185.35 ± 8.81	155.61 ± 11.26	16.04	0.08
Relative umbilical plasma flow (mL/min/kg fetus)	123.17 ± 8.27	101.08 ± 12.74	17.93	0.20
Umbilical blood flow/100 g placenta	163.27 ± 14.51	123.84 ± 13.63	24.15	0.09
Uterine: umbilical blood flow	2.68 ± 0.37	2.58 ± 0.37	3.74	0.85
Average umbilical arterial hematocrit	0.34 ± 0.02	0.36 ± 0.04	6.25	0.65
Average uterine arterial hematocrit	0.30 ± 0.01	0.31 ± 0.02	2.94	0.64

Data are shown as means ± SEM for all pregnancies in each treatment group. CSH, chorionic somatomammotropin; RNAi, RNA interference.

**Table 3:** Fetal weights, growth parameters, and uteroplacental characteristics assessed at necropsy (130 dGA).

	CON RNAi	CSH RNAi	% Change	*p*-Value
	(*n* = 4)	(*n* = 4)		
Crown-rump length, cm	49.98 ± 1.67	45.73 ± 2.10	8.50	0.16
Ponderal index	3.19 ± 0.15	2.86 ± 0.24	10.48	0.28
Lower leg length, cm	37.25 ± 1.16	32.38 ± 1.15	13.09	0.02
Brain, g	47.71 ± 1.21	46.05 ± 3.72	3.48	0.69
Brain: fetal weight	0.0121 ± 0.000	0.0172 ± 0.002	42.63	0.03
Liver, g	100.33 ± 9.13	65.50 ± 15.80	34.71	0.10
Brain: liver	0.45 ± 0.03	0.79 ± 0.21	73.65	0.17
Liver: fetal weight	0.0267 ± 0.001	0.0247 ± 0.004	7.64	0.62
Left liver lobe, g	27.19 ± 4.53	17.74 ± 4.07	34.76	0.17
Right liver lobe, g	75.46 ± 3.24	47.88 ± 11.71	36.54	0.06
Heart, g	22.48 ± 1.79	17.28 ± 2.14	23.15	0.11
Heart: fetal weight	0.0056 ± 0.000	0.0063 ± 0.001	12.29	0.23
Left ventricle, g	9.02 ± 0.81	6.68 ± 0.48	25.91	0.05
Right ventricle, g	4.99 ± 0.32	3.81 ± 0.70	23.56	0.18
Lungs, g	123.18 ± 8.76	101.61 ± 15.44	17.51	0.27
Lungs: fetal weight	0.031 ± 0.001	0.0364 ± 0.001	17.31	0.01
Pancreas, g	3.25 **±** 0.33	2.40 ± 0.42	26.21	0.16
Kidneys, g	20.05 ± 1.21	15.61 ± 2.33	22.17	0.14
Perirenal adipose tissue (PRAT), g	13.36 ± 1.96	10.58 ± 1.23	20.80	0.27
Spleen, g	6.48 ± 0.58	5.24 ± 1.24	19.24	0.40
Adrenal glands, g	0.32 ± 0.02	0.34 ± 0.06	4.38	0.86
Biceps femoris (BF), g	28.69 ± 1.22	17.97 ± 2.19	37.35	0.01
Soleus, g	0.35 ± 0.06	0.14 ± 0.04	60.28	0.02
Flexor digitorum superficialis (FDS), g	3.08 ± 0.27	1.93 ± 0.31	37.45	0.03
Tibialis anterior (TA), g	3.8 ± 0.35	2.37 ± 0.32	37.59	0.02
Extensor digitorum longus (EDL), g	1.01 ± 0.10	0.59 ± 0.10	42.22	0.03
Uteroplacental weight, g	1829.40 ± 136.67	1333.65 ± 207.93	27.10	0.09
Membrane weight, g	483.55 ± 32.18	470.28 ± 112.73	2.75	0.91
Total placentome, #	67.25 ± 4.09	71.75 ± 10.09	6.69	0.69

Data are shown as means ± SEM for all pregnancies in each treatment group. CSH, chorionic somatomammotropin; RNAi, RNA interference.

**Table 4:** In vivo measurements of nutrient transfer and uptake based on the ^3^H_2_0 transplacental diffusion technique.

130 dGA Nutrient Uptakes	CON RNAi	CSH RNAi	% Change	*p*-Value
	(*n* = 4)	(*n* = 4)		
Relative umbilical oxygen uptake (mmol/min/kg fetus)	0.33 ± 0.01	0.29 ± 0.02	12.25	0.07
Relative uterine oxygen uptake (mmol/min/kg uterus)	3.18 ± 0.54	2.96 ± 0.45	6.93	0.76
Relative uteroplacental oxygen utilization (mmol/min/kg placenta)	2.78 ± 0.42	1.90 ± 0.25	31.61	0.12
Relative umbilical glucose uptake (μmol/min/kg fetus)	34.07 ± 2.98	24.75 ± 2.70	27.36	0.06
Glucose transferred per placental weight (μmol/kg/min)	301.82 ± 41.64	199.24 ± 32.59	33.99	0.10
Relative uterine glucose uptake (μmol/min/kg uterus)	515.33 ± 85.74	461.76 ± 70.98	10.39	0.65
Relative uteroplacental glucose utilization (μmol/min/kg placenta)	634.00 ± 59.81	472.72 ± 76.49	25.44	0.15
Umbilical lactate uptake (μmol/min)	123.16 ± 5.97	75.52 ± 16.11	38.68	0.03
Relative umbilical lactate uptake (μmol/min/kg fetus)	31.23 ± 1.78	26.26 ± 2.37	15.91	0.14
Uterine lactate secretion (μmol/min)	134.17 ± 1.78	−80.41 ± 154.00	159.93	0.22
Relative uterine lactate secretion (μmol/min/kg uterus)	301.02 ± 49.60	−518.48 ± 692.82	272.24	0.28
Uteroplacental lactate production (μmol/min)	257.33 ± 16.19	−4.89 ± 168.14	101.90	0.17
Relative uteroplacental lactate production (μmol/min/kg placenta)	576.83 ± 68.83	−311.58 ± 705.10	154.02	0.26

Data are shown as means ± SEM for all pregnancies in each treatment group. CSH, chorionic somatomammotropin; RNAi, RNA interference.

**Table 5:** Concentrations of maternal or fetal hormones.

130 dGA Nutrient Uptakes	CON RNAi	CSH RNAi	% Change	*p*-Value
Maternal (Uterine)	(*n* = 4)	(*n* = 4)		
Uterine artery insulin, ng/mL	0.92 ± 0.17	0.77 ± 0.10	15.92	0.49
Uterine artery IGF1, ng/mL	297.91 ± 45.18	260.24 ± 25.74	12.64	0.50
Uterine artery cortisol, ng/mL	126.53 ± 12.95	96.27 ± 29.07	23.91	0.38
Uterine vein estradiol, pg/mL	7.32 ± 2.34	4.42 ± 1.81	39.71	0.36
**Fetal (Umbilical)**	**(*n* = 4)**	**(*n* = 4)**		
Umbilical artery insulin, ng/mL	1.12 ± 0.15	0.69 ± 0.23	38.63	0.16
Umbilical artery IGF1, ng/mL	140.89 ± 9.30	72.85 ± 14.94	48.29	0.01
Umbilical artery cortisol, ng/mL	11.98 ± 4.14	58.62 ± 29.83	389.25	0.17
Umbilical vein estradiol, pg/mL	5.59 ± 1.99	6.59 ± 0.75	17.99	0.65

Data are shown as means ± SEM for all pregnancies in each treatment group. CSH, chorionic somatomammotropin; RNAi, RNA interference.

## Data Availability

Not applicable.

## Supplementary Materials

The following are available online at https://www.mdpi.com/article/10.3390/ijms22158150/s1, Table S1: Maternal (uterine) blood gas measurements, Table S2: Fetal (umbilical) blood gas measurements, Table S3: Relative uterine uptake of individual amino acids (μmol/min/kg uterus), Table S4: Relative umbilical uptake of individual amino acids (μmol/min/kg fetus), Table S5: Relative placental uptake of individual amino acids (μmol/min/kg placenta), Table S6: Total nutrient uptakes (μmol/min), Table S7: Scramble control and CSH-targeting shRNA sequences, Figure S1: Densiometric analysis of CSH in cotyledons, Figure S2: Densiometric analysis of NOS3 in cotyledons and caruncles, Figures S3 and S4: Densiometric analysis of SLC2A1 and SLC2A3 in caruncles and cotyledons.
