# Supplementary material for: CSH RNA Interference Reduces Global Nutrient Uptake and Umbilical Blood Flow Resulting in Intrauterine Growth Restriction
Source: Int J Mol Sci. 2021 Jul 29;22(15):8150. doi: 10.3390/ijms22158150 (PMC8348624; doi:10.3390/ijms22158150)
Supplement: Supplementary file 1 [file ijms-22-08150-s001.zip › ijms-1283045-supplementary.pdf]

Supplemental Table S1. Maternal (uterine) blood gas measurements.

	CON RNAi	CSH RNAi	% change	P-value
**Uterine Artery (A)**	**(n = 4)**	**(n = 4)**		
pH $^{37.9C}$	7.458 ± 0.012	7.457 ± 0.009	0.01	0.96
pCO_2 $^{37.9C}$ mmHg	36.456 ± 1.877	35.375 ± 1.243	2.97	0.65
pO_2 $^{37.9C}$ mmHg	88.875 ± 0.704	90.219 ± 1.633	1.51	0.48
tCO_2 (B) mmol/L	22.669 ± 0.686	21.950 ± 0.725	3.17	0.50
tCO_2 (P) mmol/L	25.744 ± 0.814	24.981 ± 0.644	2.96	0.49
HCO^{3}-	24.744 ± 0.776	24.006 ± 0.621	2.98	0.49
Hct %	30.556 ± 0.593	31.088 ± 1.689	1.74	0.78
tHb mmol/L	6.131 ± 0.123	6.244 ± 0.353	1.83	0.77
O_2 Hb %	90.863 ± 0.929	91.988 ± 1.112	1.24	0.47
SO_2 %	96.363 ± 0.425	96.900 ± 1.322	0.56	0.71
CO Hb %	3.831 ± 0.092	4.106 ± 0.154	7.18	0.18
Met Hb %	1.244 ± 0.158	0.956 ± 0.066	23.12	0.14
O_2 ct mmol/L	5.700 ± 0.099	5.838 ± 0.332	2.41	0.71
Na^+ meq/L	146.438 ± 2.401	147.750 ± 0.595	0.90	0.61
K^+ meq/L	4.131 ± 0.123	4.419 ± 0.101	6.96	0.12
Ca^{2+} meq/L	2.734 ± 0.033	2.776 ± 0.029	1.53	0.38
**Uterine Vein (V)**	**(n = 4)**	**(n = 4)**		
pH $^{37.9C}$	7.446 ± 0.015	7.424 ± 0.008	0.30	0.24
pCO_2 $^{37.9C}$ mmHg	40.025 ± 1.280	40.275 ± 1.227	0.62	0.89
pO_2 $^{37.9C}$ mmHg	54.675 ± 1.422	54.281 ± 1.342	0.72	0.85
tCO_2 (B) mmol/L	23.731 ± 0.635	23.300 ± 0.689	1.82	0.66
tCO_2 (P) mmol/L	26.769 ± 0.763	26.325 ± 0.579	1.66	0.66
HCO^{3}-	25.681 ± 0.731	25.200 ± 0.559	1.87	0.62
Hct %	30.756 ± 0.621	31.213 ± 1.746	1.48	0.81
tHb mmol/L	6.169 ± 0.123	6.256 ± 0.362	1.42	0.83
O_2 Hb %	69.319 ± 2.116	69.775 ± 3.772	0.66	0.92
SO_2 %	72.131 ± 2.239	72.631 ± 4.144	0.69	0.92
CO Hb %	2.706 ± 0.112	2.994 ± 0.304	10.62	0.41
Met Hb %	1.163 ± 0.126	0.906 ± 0.077	22.04	0.13
O_2 ct mmol/L	4.325 ± 0.096	4.456 ± 0.419	3.03	0.77
Na^+ meq/L	147.500 ± 2.588	149.000 ± 1.177	1.02	0.62
K^+ meq/L	4.131 ± 0.103	4.344 ± 0.138	5.14	0.26
Ca^{2+} meq/L	2.704 ± 0.030	2.718 ± 0.016	0.53	0.68

Data are shown as means ± SEM for all pregnancies in each treatment group. CSH, chorionic somatomammotropin; RNAi, RNA interference.

Supplemental Table S2. Fetal (umbilical) blood gas measurements.

	CON RNAi	CSH RNAi	% change	P-value
**Umbilical artery (α)**	**(n = 4)**	**(n = 4)**		
pH $^{37.9C}$	7.346 ± 0.004	7.352 ± 0.006	0.08	0.46
pCO_2 $^{37.9C}$ mmHg	51.313 ± 1.758	52.106 ± 1.470	1.55	0.74
pO_2 $^{37.9C}$ mmHg	21.238 ± 0.241	18.031 ± 2.066	15.10	0.17
tCO_2 (B) mmol/L	24.850 ± 0.979	25.369 ± 0.556	2.09	0.66
tCO_2 (P) mmol/L	27.988 ± 0.994	28.819 ± 0.864	2.97	0.55
HCO^{3-}	26.575 ± 0.940	27.350 ± 0.807	2.92	0.55
Hct %	33.144 ± 1.807	35.713 ± 4.117	7.75	0.59
tHb mmol/L	6.800 ± 0.308	7.181 ± 0.855	5.61	0.69
O_2 Hb %	49.525 ± 1.660	40.200 ± 7.859	18.83	0.29
SO_2 %	51.856 ± 1.367	41.288 ± 7.758	20.38	0.23
CO Hb %	1.931 ± 0.108	1.500 ± 7.758	22.33	0.19
Met Hb %	0.250 ± 0.071	0.338 ± 0.161	35.00	0.64
O_2 ct mmol/L	3.538 ± 0.145	2.719 ± 0.345	23.14	0.07
Na^+ meq/L	141.813 ± 2.796	143.688 ± 1.028	1.32	0.55
K^+ meq/L	3.463 ± 0.155	3.913 ± 0.189	13.00	0.12
Ca^{2+} meq/L	2.977 ± 0.050	2.899 ± 0.104	2.62	0.52
**Umbilical vein (γ)**	**(n = 4)**	**(n = 4)**		
pH $^{37.9C}$	7.388 ± 0.004	7.392 ± 0.003	0.06	0.46
pCO_2 $^{37.9C}$ mmHg	43.588 ± 1.503	44.750 ± 1.656	2.67	0.62
pO_2 $^{37.9C}$ mmHg	32.881 ± 0.484	27.406 ± 3.772	16.65	0.20
tCO_2 (B) mmol/L	23.369 ± 1.204	23.688 ± 0.602	1.36	0.82
tCO_2 (P) mmol/L	26.225 ± 0.961	27.175 ± 0.890	3.62	0.50
HCO^{3-}	25.013 ± 0.925	25.938 ± 0.854	3.70	0.49
Hct %	33.494 ± 1.471	35.419 ± 4.151	5.75	0.68
tHb mmol/L	6.744 ± 0.310	7.125 ± 0.857	5.65	0.69
O_2 Hb %	79.244 ± 0.961	68.063 ± 9.995	14.11	0.31
SO_2 %	82.281 ± 1.159	69.963 ± 10.365	14.97	0.28
CO Hb %	3.500 ± 0.448	2.656 ± 0.353	24.11	0.19
Met Hb %	0.106 ± 0.232	0.031 ± 0.165	70.59	0.80
O_2 ct mmol/L	5.350 ± 0.187	4.613 ± 0.308	13.79	0.09
Na^+ meq/L	142.375 ± 2.260	144.063 ± 1.625	1.19	0.57
K^+ meq/L	3.463 ± 0.155	3.925 ± 0.193	13.36	0.11
Ca^{2+} meq/L	3.050 ± 0.057	2.984 ± 0.107	2.15	0.61

Data are shown as means ± SEM for all pregnancies in each treatment group. CSH, chorionic somatomammotropin; RNAi, RNA interference.

Supplemental Table S3. Relative uterine uptake of individual amino acids (μmol/min/kg uterus).

	CON RNAi	CSH RNAi	% change	P-value
**Amino acid**	**(n = 4)**	**(n = 4)**		
Tau	0.07 ± 0.66	-2.42 ± 1.19	3560.98	0.12
Asp	-1.52 ± 0.38	-0.99 ± 0.77	34.63	0.56
Thr	16.29 ± 3.36	10.36 ± 2.19	36.44	0.19
Ser	15.98 ± 2.82	11.37 ± 1.04	28.87	0.18
Asn	5.97 ± 1.52	4.07 ± 0.64	31.80	0.29
Glu	-1.53 ± 0.37	-2.46 ± 1.26	60.33	0.51
Gln	30.40 ± 5.60	20.94 ± 3.47	31.12	0.20
Pro	10.13 ± 1.68	10.29 ± 4.16	1.50	0.97
Gly	-6.90 ± 3.84	-14.25 ± 4.72	106.61	0.27
Ala	11.99 ± 2.25	4.65 ± 1.95	61.27	0.05
Cit	10.72 ± 2.15	9.38 ± 2.25	12.51	0.68
Val	29.40 ± 4.93	23.30 ± 4.84	20.74	0.41
Cys	2.21 ± 1.41	-0.02 ± 0.57	100.94	0.19
Met	3.32 ± 0.77	1.92 ± 1.04	42.05	0.32
Ile	17.82 ± 3.60	12.12 ± 2.28	32.00	0.23
Leu	23.76 ± 4.22	18.33 ± 3.43	22.86	0.36
Tyr	4.74 ± 1.04	2.19 ± 0.84	53.69	0.11
Phe	4.70 ± 1.19	1.75 ± 0.60	62.85	0.07
Trp	2.44 ± 0.85	2.81 ± 0.64	14.98	0.74
Orn	11.37 ± 1.83	11.97 ± 0.81	5.28	0.77
Lys	15.49 ± 2.70	7.25 ± 1.71	53.15	0.04
His	5.85 ± 1.51	3.78 ± 0.95	35.43	0.29
Arg	17.97 ± 4.36	8.42 ± 3.08	53.12	0.12

Data are shown as means ± SEM for all pregnancies in each treatment group. CSH, chorionic somatomammotropin; RNAi, RNA interference.

Supplemental Table S4. Relative umbilical uptakes of individual amino acids (μmol/min/kg fetus).

	CON RNAi	CSH RNAi	% change	P-value
**Amino acid**	**(n = 4)**	**(n = 4)**		
Tau	-0.03 ± 0.09	-0.42 ± 0.17	1474.97	0.08
Asp	-0.09 ± 0.10	-0.05 ± 0.10	47.20	0.78
Thr	2.58 ± 0.20	2.30 ± 0.93	10.64	0.78
Ser	-0.41 ± 0.34	-0.34 ± 1.03	18.19	0.95
Asn	1.61 ± 0.10	1.21 ± 0.17	24.50	0.09
Glu	-3.12 ± 0.21	-2.12 ± 0.60	32.00	0.17
Gln	8.58 ± 0.46	6.76 ± 1.25	21.22	0.22
Pro	1.77 ± 0.45	1.69 ± 0.43	4.70	0.90
Gly	3.38 ± 0.23	3.06 ± 1.02	9.44	0.77
Ala	4.25 ± 0.17	3.16 ± 0.83	25.61	0.24
Cit	0.42 ± 0.24	0.29 ± 0.30	30.20	0.75
Val	4.66 ± 0.15	3.98 ± 0.95	14.55	0.51
Cys	0.13 ± 0.03	0.06 ± 0.13	56.42	0.61
Met	0.96 ± 0.08	0.69 ± 0.25	27.96	0.35
Ile	2.76 ± 0.14	2.21 ± 0.35	19.95	0.20
Leu	4.44 ± 0.19	3.60 ± 0.51	18.94	0.17
Tyr	1.57 ± 0.09	1.21 ± 0.19	23.03	0.14
Phe	1.61 ± 0.12	1.29 ± 0.29	19.65	0.35
Trp	0.39 ± 0.08	0.19 ± 0.13	49.91	0.25
Orn	0.15 ± 0.10	0.13 ± 0.25	14.38	0.94
Lys	2.71 ± 0.27	2.36 ± 0.40	12.65	0.50
His	0.75 ± 0.05	0.57 ± 0.16	24.76	0.30
Arg	2.77 ± 0.28	2.37 ± 0.49	14.52	0.50

Data are shown as means ± SEM for all pregnancies in each treatment group. CSH, chorionic somatomammotropin; RNAi, RNA interference.

Supplemental Table S5. Relative uteroplacental utilization of individual amino acids (μmol/min/kg placenta).

	CON RNAi	CSH RNAi	% change	P-value
**Amino acid**	**(n = 4)**	**(n = 4)**		
Tau	0.82 ± 1.35	0.00 ± 1.47	99.92	0.70
Asp	-2.23 ± 1.05	-1.12 ± 0.59	49.45	0.40
Thr	6.23 ± 5.11	-2.25 ± 4.30	136.04	0.25
Ser	32.36 ± 5.48	21.78 ± 9.55	32.69	0.37
Asn	-3.65 ± 2.15	-3.66 ± 0.81	0.21	1.00
Glu	24.37 ± 2.19	14.20 ± 4.39	41.73	0.08
Gln	-20.26 ± 5.79	-21.92 ± 5.62	8.21	0.84
Pro	4.24 ± 3.89	2.48 ± 7.55	41.42	0.84
Gly	-41.92 ± 6.74	-41.64 ± 10.39	0.68	0.98
Ala	-15.00 ± 3.35	-17.92 ± 2.59	19.49	0.52
Cit	15.80 ± 3.08	11.57 ± 3.74	26.79	0.42
Val	12.29 ± 4.46	4.16 ± 8.06	66.14	0.41
Cys	2.58 ± 2.32	-0.32 ± 2.01	112.54	0.38
Met	-2.38 ± 1.50	-2.71 ± 1.06	13.85	0.86
Ile	7.29 ± 3.35	0.04 ± 1.67	99.50	0.10
Leu	3.21 ± 4.46	-2.15 ± 2.16	166.78	0.32
Tyr	-4.84 ± 1.11	-6.67 ± 0.68	37.75	0.21
Phe	-5.74 ± 2.00	-7.55 ± 0.72	31.52	0.43
Trp	0.83 ± 1.16	2.65 ± 0.78	218.76	0.24
Orn	20.08 ± 3.31	17.57 ± 4.34	12.51	0.66
Lys	4.01 ± 3.20	-7.52 ± 2.62	287.65	0.03
His	3.43 ± 1.93	1.04 ± 1.30	69.56	0.35
Arg	9.06 ± 6.75	-3.45 ± 6.33	138.10	0.22

Data are shown as means ± SEM for all pregnancies in each treatment group. CSH, chorionic somatomammotropin; RNAi, RNA interference.

Supplemental Table S6. Total nutrient uptakes (µmol/min)

	CON RNAi	CSH RNAi	% change	P-value
**Uterine**	**(n = 4)**	**(n = 4)**		
Sum of uterine amino acid carbon uptake	1006.29 ± 112.94	405.94 ± 71.71	59.66	0.004
Sum of uterine glucose carbon uptake	2562.06 ± 340.25	1412.67 ± 266.44	44.86	0.04
Sum of lactate carbon uptake	402.52 ± 56.07	-241.24 ± 462.00	159.93	0.22
Sum of uterine carbon uptake	3970.88 ± 491.12	1577.36 ± 783.96	60.28	0.04
Sum of uterine nitrogen uptake	318.94 ± 28.48	123.25 ± 18.19	61.36	0.001
**Umbilical**	**(n = 4)**	**(n = 4)**		
Sum of umbilical amino acid carbon uptake	840.11 ± 65.12	512.89 ± 172.70	38.95	0.13
Sum of umbilical glucose carbon uptake	807.81 ± 75.88	430.07 ± 96.37	46.76	0.02
Sum of umbilical lactate carbon uptake	369.47 ± 17.90	226.57 ± 48.32	38.68	0.03
Sum of total fetal carbon uptake	2017.39 ± 113.97	1169.53 ± 295.20	42.03	0.04
Sum of umbilical nitrogen uptake	262.04 ± 19.78	159.64 ± 54.50	39.08	0.13
Umbilical glucose:oxygen quotient	0.61 ± 0.06	0.50 ± 0.04	17.88	0.15
Umbilical lactate:oxygen quotient	0.28 ± 0.01	0.27 ± 0.01	4.58	0.46
Umbilical amino acid:oxygen quotient	0.68 ± 0.03	0.63 ± 0.11	7.96	0.66
Total umbilical nutrient quotient	1.58 ± 0.06	1.40 ± 0.12	11.22	0.24

Data are shown as means ± SEM for all pregnancies in each treatment group. CSH, chorionic somatomammotropin; RNAi, RNA interference.

Supplemental Table S7. Scrambled control and CSH-targeting shRNA sequences.

Oligonucleotide	Sequence (5′-3′)
NTS shRNA sense	GAGTTAAAGGTTCGGCACGAATTCAAGAGATTCGTGCCGAACCTTTAACTC
tg6 shRNA sense	AAGGCCAAAGTACTTGTAGACTTCAAGAGAGTCTACAAGTACTTTGGCCTT

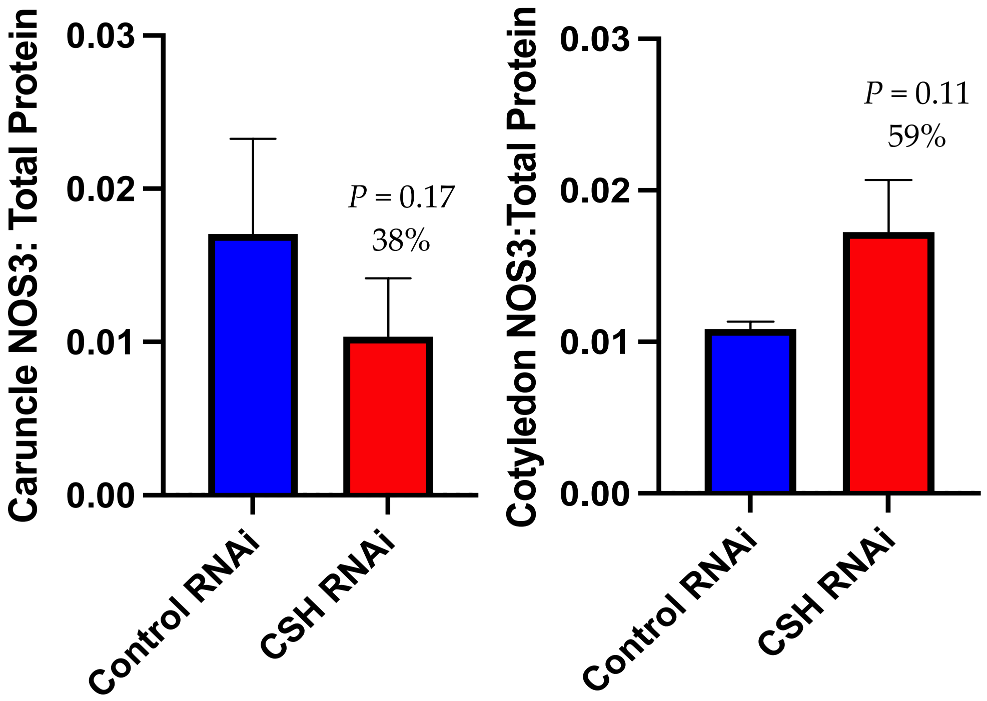

Supplemental Figure S1. Densiometric analysis of NOS3 relative to total protein transferred, in fetal cotyledons from control RNA interference (RNAi) and chorionic somatomammotropin (CSH) RNAi pregnancies (n = 4/treatment). Data are shown as means ± SEM for all pregnancies in each treatment group. CSH, chorionic somatomammotropin; RNAi, RNA interference.

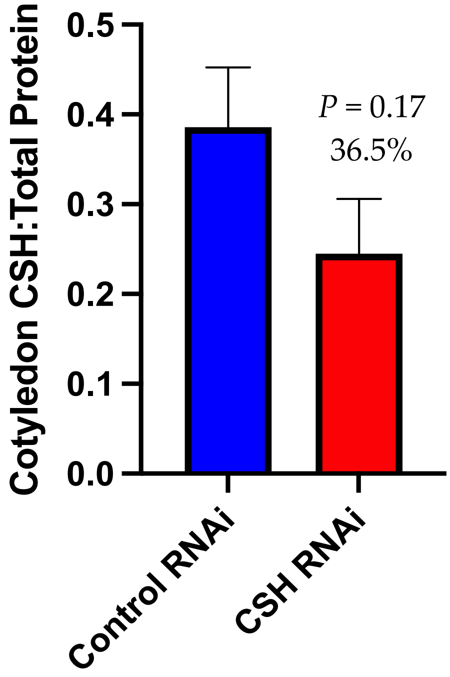

Supplemental Figure S2. Densiometric analysis of CSH, relative to total protein transferred, in fetal cotyledons from control RNA interference (RNAi) and chorionic somatomammotropin (CSH) RNAi pregnancies (n = 4/treatment). Data are shown as means ± SEM for all pregnancies in each treatment group. CSH, chorionic somatomammotropin; RNAi, RNA interference.

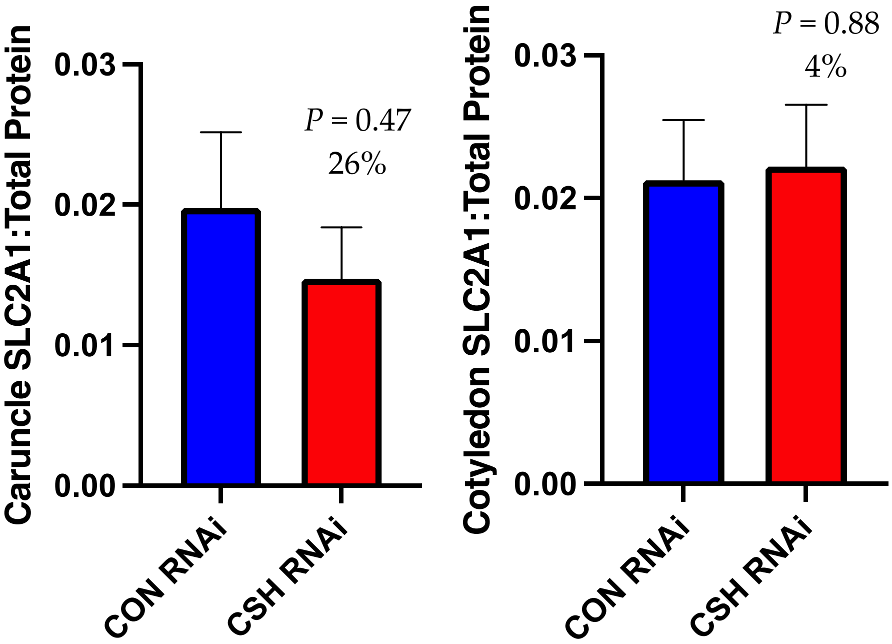

Supplemental Figure S3. Densiometric analysis of SLC2A1 relative to total protein transferred, in fetal cotyledons from control RNA interference (RNAi) and chorionic somatomammotropin (CSH) RNAi pregnancies (n = 4/treatment). Data are shown as means ± SEM for all pregnancies in each treatment group. CSH, chorionic somatomammotropin; RNAi, RNA interference.

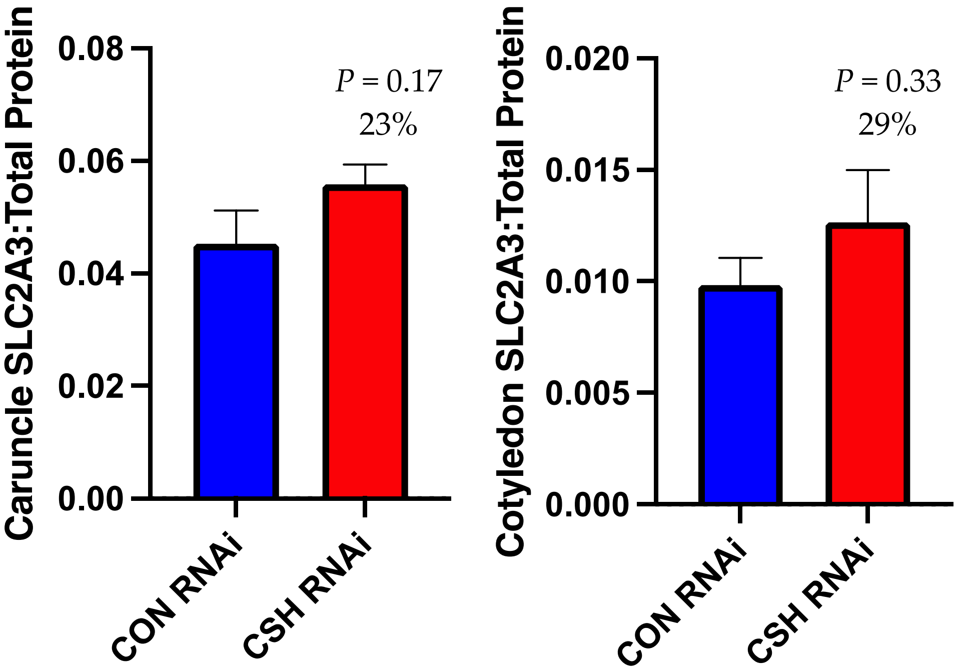

Supplemental Figure S4. Densiometric analysis of SLC2A3 relative to total protein transferred, in fetal cotyledons from control RNA interference (RNAi) and chorionic somatomammotropin (CSH) RNAi pregnancies (n = 4/treatment). Data are shown as means ± SEM for all pregnancies in each treatment group. CSH, chorionic somatomammotropin; RNAi, RNA interference.
